# Ultrasound and shear-wave elastography patterns of COVID-19 mRNA vaccine-related axillary, supra and subclavicular lymphadenopathy

**DOI:** 10.1007/s40336-021-00441-0

**Published:** 2021-06-18

**Authors:** Divina D’Auria, Ludovica Fulgione, Valeria Romeo, Arnaldo Stanzione, Simone Maurea, Arturo Brunetti

**Affiliations:** Department of Advanced Biomedical Sciences, University of Naples “Federico II”, Via S. Pansini, 5, 80131 Naples, Italy

**Keywords:** Covid-19, Lymphadenopathy, Ultrasound, Shear-wave elastography, Vaccine, mRNA vaccine

## Abstract

In this pictorial essay, we illustrate the ultrasound appearance of COVID-19 Pfizer-BioNTech vaccine-related lymph node abnormalities, which can occur at different stations ipsilateral to the site of vaccination, after either first or second vaccine dose and can represent a diagnostic dilemma when encountered in patients with underlying conditions. Typically, they appear as enlarged hypoechoic nodes with loss of fat hilum, increased hilar and cortical vascularization at color-Doppler, but low to intermediate cortical consistence at shear-wave elastography. Asymmetric or diffuse cortical thickening is also frequently encountered. They can be observed in patients without and with clinical symptoms, such as armpit pain, fever and fatigue.

## Introduction

Achieving herd immunity through a mass vaccination campaign currently represents the most promising strategy in the pursuit of an end to the global crisis related to the severe acute respiratory syndrome coronavirus 2 (SARS-CoV-2) pandemic, as demonstrated in Israel by the recent results achieved with the BNT162b2 mRNA (Pfizer-BioNTech) vaccine for coronavirus disease 2019 (COVID-19) [1].This encouraging evidence confirms the clinical trials findings which had also revealed a reassuring safety profile of the Pfizer-BioNTech vaccine, with mostly minor and transient side effects reported [2]. Among these, ipsilateral vaccine-related lymphadenopathy is expected to become a common incidental imaging finding to be dealt with, while the data regarding their number, size, sites, morphology and imaging features are still scarce [3]. In oncologic patients, these abnormal findings might be mistaken for metastases and a recent history of COVID-19 vaccine can provide relevant information to guide the differential diagnosis of enlarged lymph nodes [4, 5]. Ultrasound (US) is a widespread and extensively used imaging techniques and particularly the first-choice modality for lymph nodes assessment. We, therefore, aim to provide an overview of the most relevant US findings of COVID-19 vaccine encountered in health worker subjects, including B-mode, color-Doppler and shear-wave elastography of both axillary and supra/subclavicular lymph nodes, also trying to define a temporal evolution of detected lymphadenopathies trough a 1-month follow-up assessment.

## Cases presentation

US examinations were performed in young healthcare professionals, with no previous pathological conditions, between February and March 2021 during the Italian vaccination campaign, who presented at our Radiology Department after receiving Pfizer-BioNTech vaccine. US were performed using a GE Logiq S8 scanner equipped with a high-frequency linear probe (11–15 MHz). Bilateral axillary, supraclavicular, sub-clavicular and cervical lymph node stations were explored. A first B-mode evaluation was performed with measurement of long/short lymph node axis as well as cortical thickness. Lymph node shape, margins and fatty hilum were also evaluated. The examination was completed with color-Doppler assessment of hilar/cortical vascularization and quantitative shear-wave elastography measurement of thickened cortex. In female subjects, breast US was also performed to rule out the presence of suspected/pathological breast lesions that could explain the occurrence of ipsilateral lymphadenopathies.

All subjects gave their written informed consent for the use of both imaging and clinical data for research purposes.

### Case 1

**Fig. 1:**
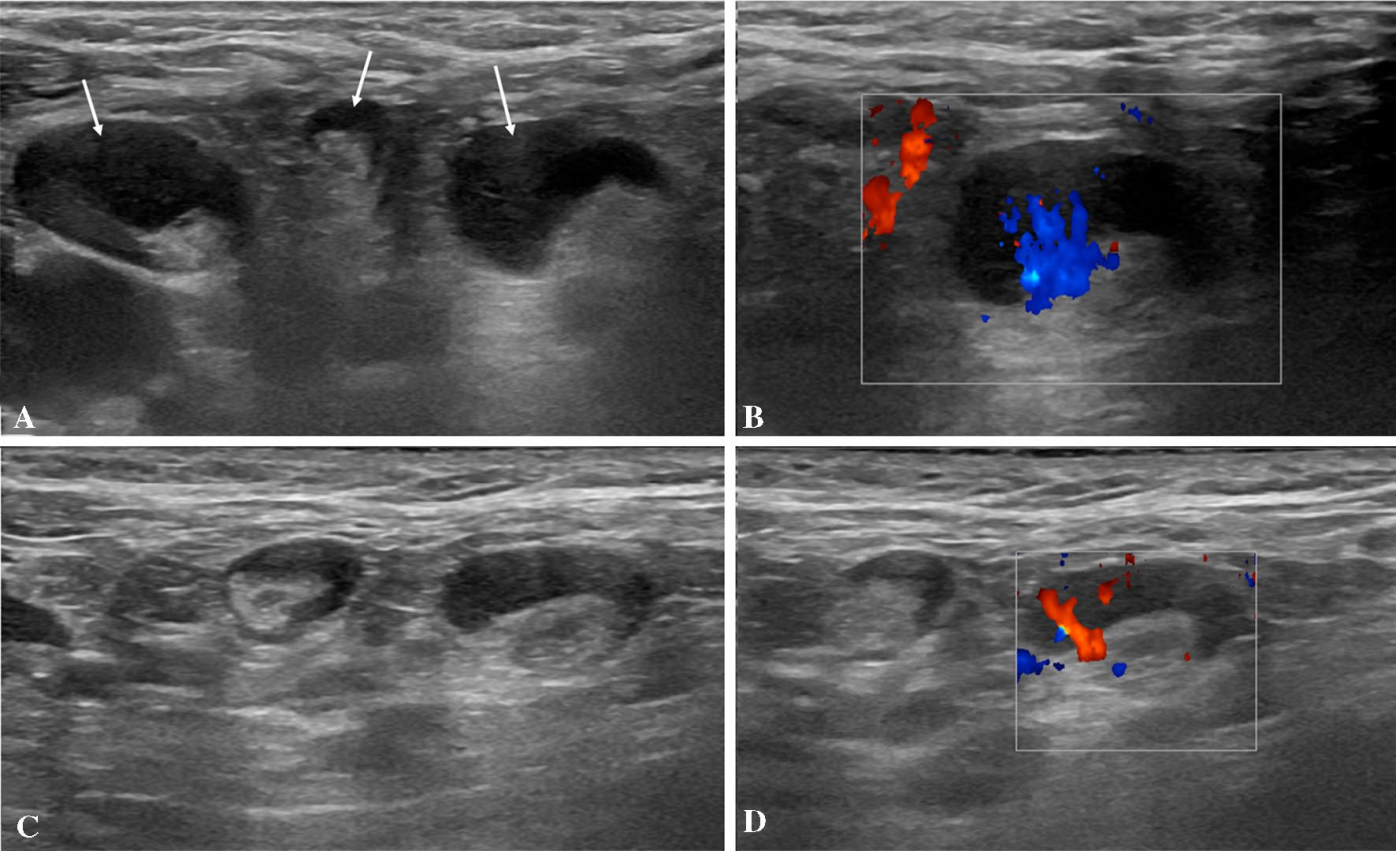
B-Mode (**A**) and color-Doppler (**B**) US examination performed in a 28-year-old woman 2 days after receiving the second dose of vaccine: axillary lymph nodes (maximum diameter 14 × 9 mm) with fatty hilum but diffuse cortical thickening (maximum 10 mm, arrows in **A**) and vivid hilar and cortical vascularization (**B**) were detected. B-Mode (**C**) and color-Doppler (**D**) US examination performed after 4 weeks: reduction of lymph nodes size (7 × 6 mm), cortical thickness (2.9 mm) and vascularization was observed

A 28-year-old female subject underwent US examination 2 days after receiving the second dose of Pfizer-BioNTech vaccine due to palpable left axillary lump and ipsilateral armpit pain (same side of vaccine injection). She also experienced fatigue and headache. US showed the presence of axillary lymph nodes (maximum diameter 14 × 9 mm) with preserved fatty hilum, but diffuse cortical thickening (10 mm) and vivid hilar and cortical vascularization. A soft consistence of the cortex was found at shear-wave elastography (mean values between 3.4 and 8.1 kPa). No pathological lymph nodes were found in ipsilateral supra and subclavicular lymph node stations as well as in lymph nodes contralateral to the site of vaccine injection. Follow-up US examination was performed after 4 weeks and a reduction of lymph node size (7 × 6 mm), cortical thickness (2.9 mm) and vascularization was found (Fig. 1).

### Case 2

**Fig. 2:**
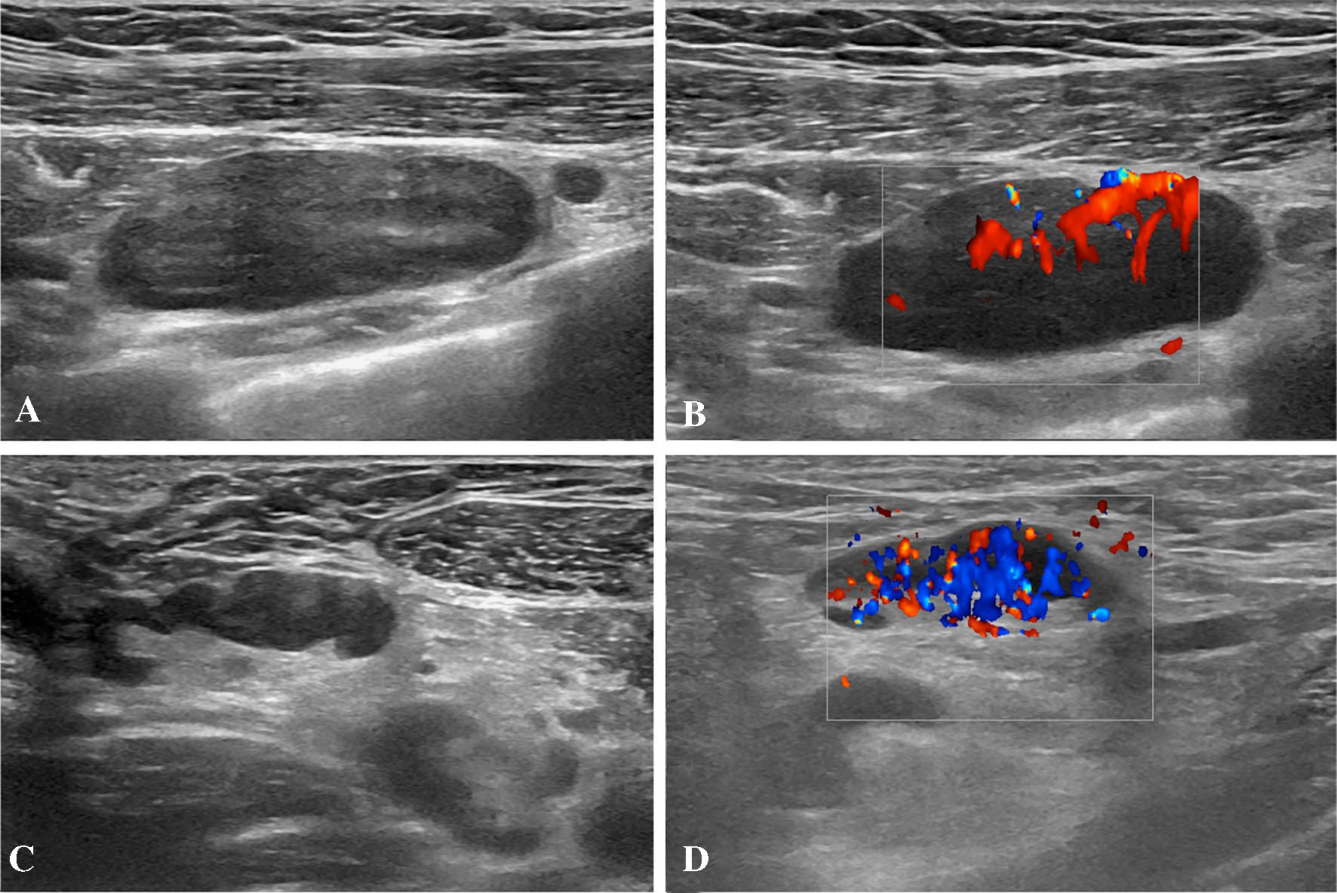
B-Mode (**A**, **B**) and color-Doppler (**B**, **D**) US examination performed in a 28-year-old woman 2 days after receiving the second dose of vaccine. Enlarged (33 × 11 mm) subclavicular lymph node with no detectable fatty hilum (**A**) and vivid hilar and cortical vascularization (**B**) was detected. Enlarged axillary lymph node (26 × 7 mm) with cortical thickening (5 mm) (**C**) and increased vascularization (**D**) were also found

A 28-year-old woman, who experienced headache and left axillary pain after the second vaccine dose, performed a US examination 2 days after that revealed the presence of enlarged (26 × 7 mm) axillary lymph nodes with preserved fatty hilum, thickened cortex (5 mm) and increased vascularization at color-Doppler. A pathological ipsilateral sub-clavicular lymph node (33 × 11 mm) was also found with no visible fatty hilum and increased vascular signal (Fig. 2). In both nodes, shear-wave elastography showed a soft texture (mean values between 13 and 18 kPa). No pathological lymph nodes were found in lymph node stations contralateral to the site of vaccine injection. The patient had previously undergone a breast US for a routine control after receiving the first vaccine dose, that did not show any significant abnormalities.

### Case 3

**Fig. 3:**
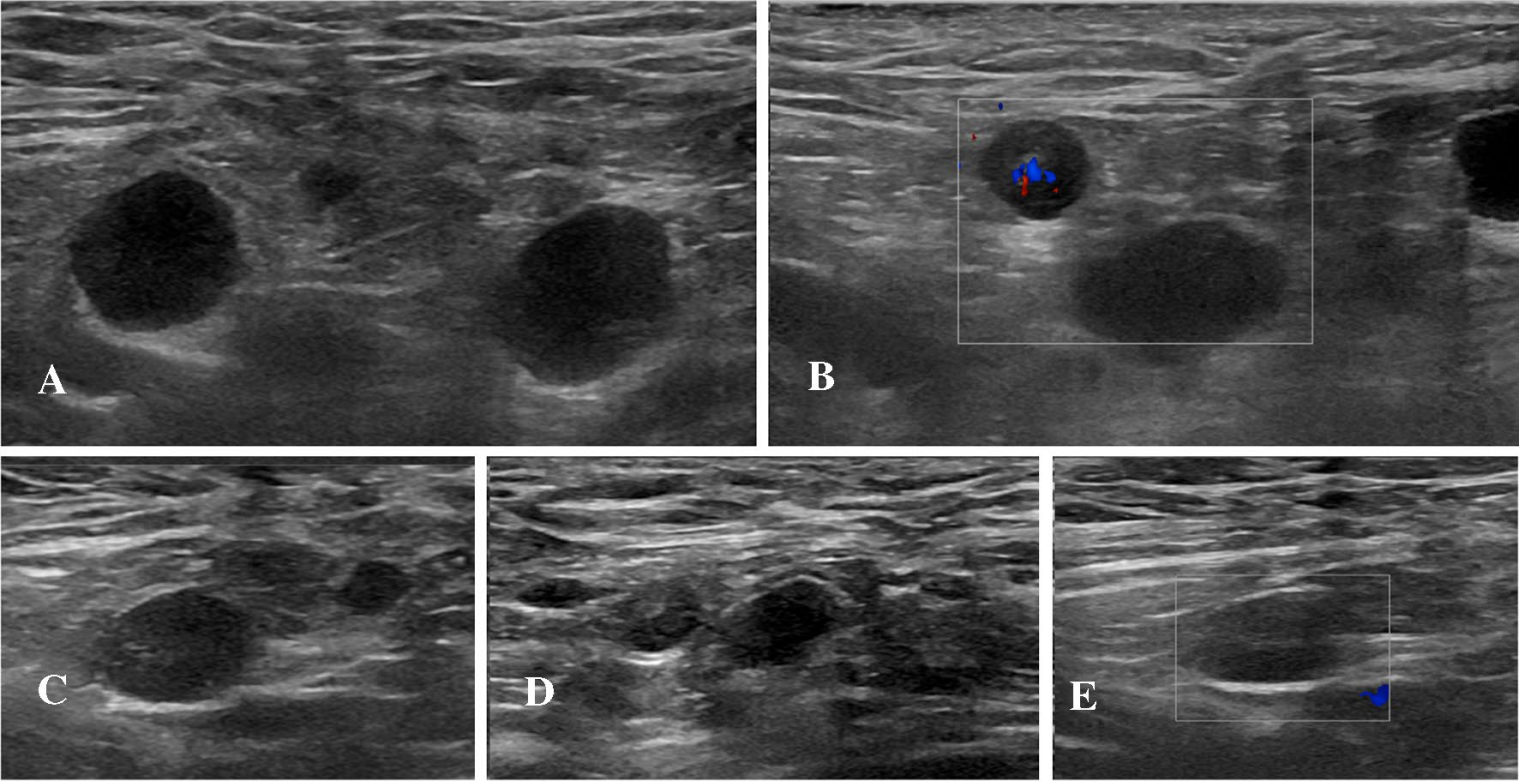
B-Mode (**A**) and color-Doppler (**B**) US examination performed 3 days after receiving the second vaccine dose. Multiple round (maximum diameter 11 × 10 mm) axillary lymph nodes with no fatty hilum and mild hilar vascularization were detected. B-Mode (**C**, **D**) and color-Doppler (**E**) US examination performed after 5 weeks. Only a reduction of lymph node size (maximum diameter 9 × 6 mm) was detectable.

A 29-year-old female subject underwent US examination 3 days after receiving the second dose of Pfizer-BioNTech vaccine, due to the rapid occurrence of a palpable lump at the level of the left axilla (same site of vaccine injection), along with fatigue. Multiple, round, hypoechoic axillary lymph nodes (maximum diameter 11 × 10 mm) with no fatty hilum and mild hilar vascularization. Shear-wave elastography revealed a soft consistence (mean values between 6 and 19 kPa). Follow-up US examination performed 5 weeks after the second dose only showed a reduction in lymph node size (maximum diameter 9 × 6 mm) (Fig. 3). No pathological lymph nodes were found in both ipsilateral and contralateral supra/subclavicular and cervical stations as well as in the contralateral axilla.

### Case 4

**Fig. 4:**
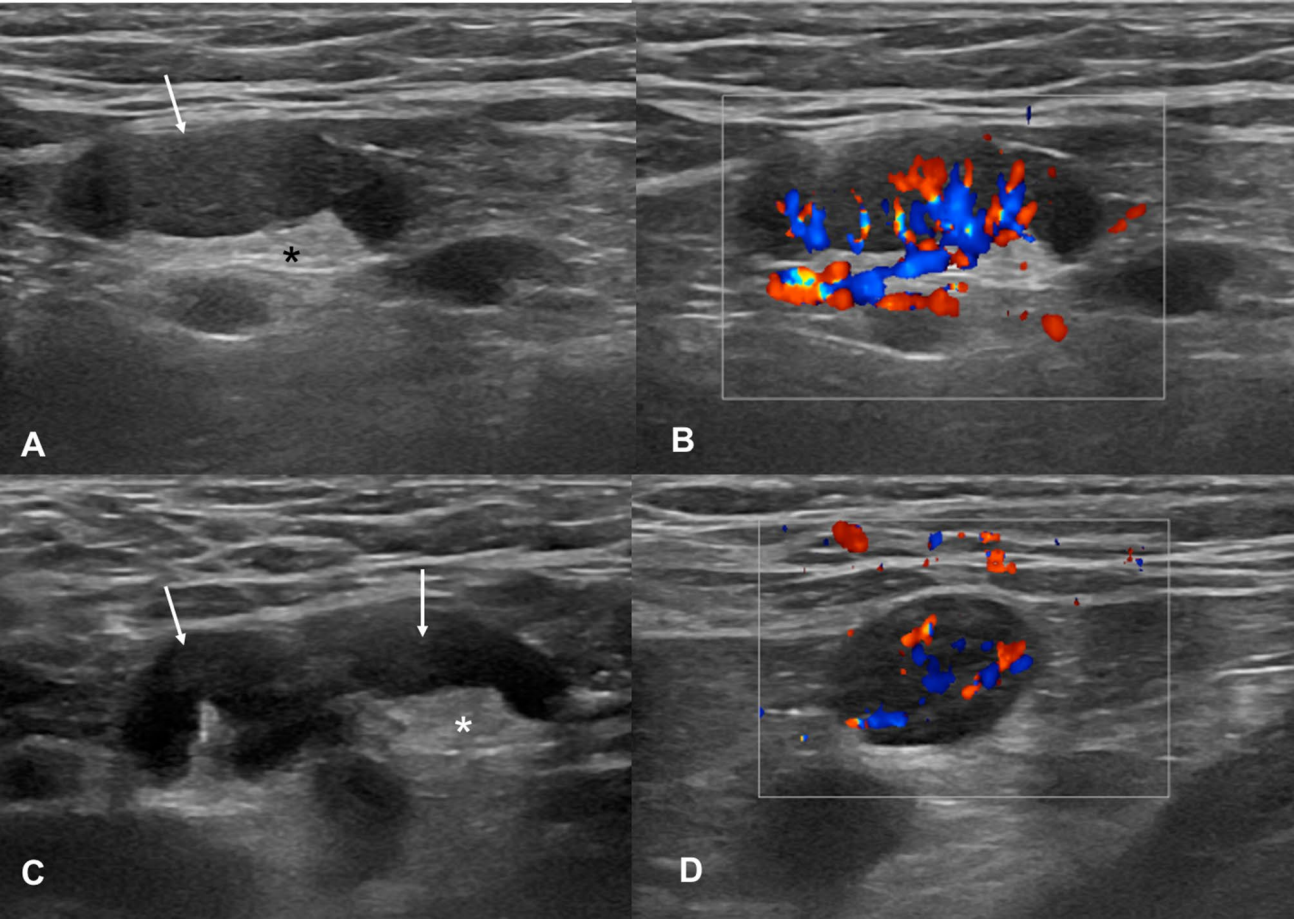
B-Mode (**A**, **C**) and color-Doppler (**B**, **D**) US examination performed 3 days after receiving the second dose of vaccine. Enlarged (maximum diameter 21 × 7 mm) axillary lymph node with preserved fatty hilum (* in **A** and **C**), diffuse cortical thickening (6 mm, arrows in **A** and **C**) and vivid hilar and cortical vascularization (**B**, **D**) were found

A 28-year-old male asymptomatic subject underwent US examination for screening purpose 3 days after the second vaccine dose. A round hypoechoic axillary lymph node (13 × 20 mm) was found with increased vascular signal at color-Doppler examination at the same site of vaccine injection (left arm). An enlarged axillary lymph node was also detected (21 × 7 mm) with preserved hilar representation but diffuse cortical thickening (6 mm) and abnormal vascularization. Both lymph nodes showed soft consistence at shear-wave sonoelastography (mean values between 27 and 38 kPa). No pathological lymph nodes were found in both ipsilateral and contralateral supra/subclavicular and cervical stations as well as in the contralateral axilla. Both findings were no longer detectable at 4 weeks US examination (Fig. 4).

### Case 5

**Fig. 5:**
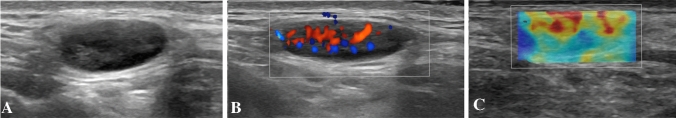
B-Mode (**A**), color-Doppler (**B**) and shear wave (**C**) US examination performed 8 days after receiving the first dose of vaccine. Enlarged (14 × 6 mm) supraclavicular lymph node with no fatty hilum (**A**), increased hilar and cortical vascularization (**B**) and soft-intermediate consistence at shear-wave elastography (**C**) is shown

US examination was performed in a 33-year-old woman with the occurrence of axillary pain and a palpable lump in the supraclavicular region 8 days after the first dose of Pfizer-BioNTech vaccine. The patient had also experienced fever and fatigue right after vaccine injection. Both axillary and supraclavicular lymph nodes were found enlarged (maximum diameter 14 × 6 mm), with no fatty hilum, increased vascularization and soft-intermediate consistence at shear-wave sonoelastography (Fig. 5). No pathological lymph nodes were found in the contralateral axilla. US lymph node features were substantially stable at US examinations performed after 3 weeks.

### Case 6

A 30-year-old woman with the onset of fever, myalgia and left armpit pain ipsilateral to vaccine injection after the first dose underwent US examination 5 days later. Multiple axillary lymphadenopathies (maximum diameter 15 × 10 mm) with round to oval shape, poor hilar representation, thickened cortex and increased vascularization at color-Doppler were found. No pathological lymph nodes were found in ipsilateral supra and subclavicular lymph node stations as well as in lymph nodes contralateral to the site of vaccine injection.

**Fig. 6:**
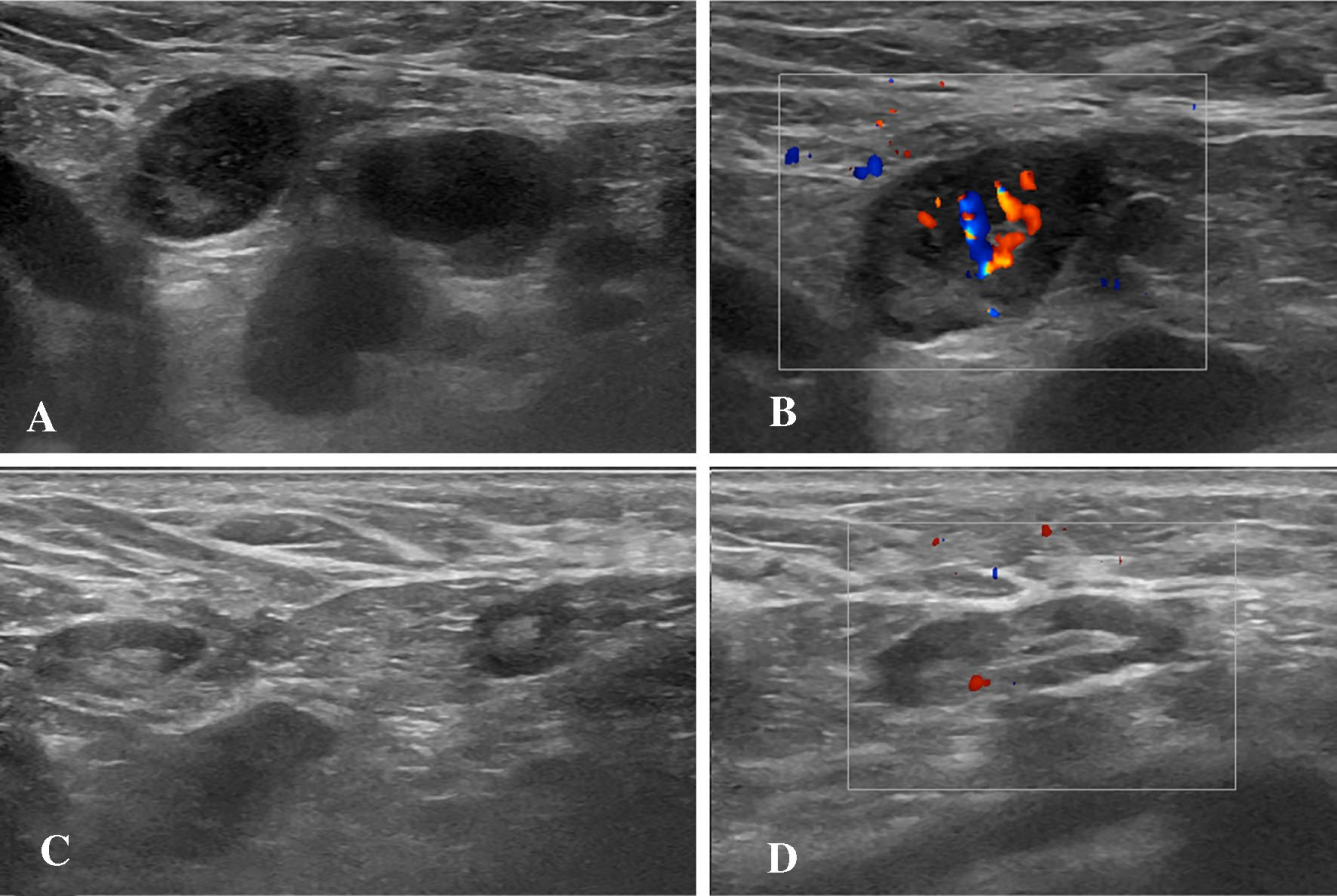
B-Mode (**A**) and color-Doppler (**B**) US examination performed 2 days after receiving the first dose of vaccine: multiple axillary lymph nodes (maximum diameter 15 × 10 mm) with poorly represented fatty hilum (**A**) and moderate vascularization (**B**) were found. B-Mode (**C**) and color-Doppler (**D**) US examination performed after 4 weeks. Although lymph node size remained stable, fatty hilum was restored (**C**) and vascularization reduced at color-Doppler (**D**)

US follow-up performed 4 weeks later showed stable lymph node size with restored fatty hilum as well as reduced cortical thickness and vascularization (Fig. 6).

## Discussion

As the world approached the large-scale COVID-19 vaccine distribution with several million doses of mRNA COVID-19 vaccines first rapidly rolled out to the population, many local and systemic reactions were observed after injections, frequently after the second dose. While axillary adenopathy has been rarely reported for other vaccinations such as BCG, influential and human papilloma vaccinations, it seems to be a very common reaction after COVID-19 vaccine [6–8]. Hyperplastic axillary nodes that can indeed be seen after COVID-19 vaccination showing heterogeneous findings in morphology including size, shape and echogenicity with no clearly described patterns. According to the Centers for Disease Control and Prevention, more than 11% of other vaccine cases lead to swollen lymph nodes after one dose, rising to 16% after the second [9]. Such symptoms usually appear 2–4 days after the vaccine and decline after 2 weeks. Based on our experience, solitary and multiple axillary, supra and subclavicular lymph nodes can be found abnormal at US examination performed after both first and second dose of Pfizer-BioNTech vaccine, ipsilaterally to the site of injection, in young healthy subjects. No pathological cervical lymph nodes were found in our case series. Five out of six subjects presented clinical symptoms, such as ipsilateral armpit pain, palpable axillary lump, fatigue and fever. However, pathological findings can also be found in asymptomatic subjects, as occurred in the remaining sixth case. Imaging features consisted of hypoechoic round or oval nodes as well as lymph node with preserved fatty hilum, but increased (> 3 mm) diffuse or asymmetric cortical thickness, thus mimicking nodal metastases. Vascular signal was mainly found to be increased, localized in both hilar and cortical regions. Shear-wave elastography showed a soft cortical consistence in all cases, with quantitative values comprised between 3.4 and 38 kPa. All values were inferior to the reported threshold for malignancy, 42 kPa, which showed a negative predictive value of 93.2 in a recent study [10]. Although this is not the first case series reporting US features of COVID-19 vaccine-related lymphadenopathies, to the best of our knowledge this is the first one focused on US findings also providing shear-wave sonoelastography data that were not provided in any of the previous experiences [11]. As for B-mode and color-Doppler imaging, cortical thickening and increased vascularity are confirmed as the key imaging features.

Based on the described findings, it is also possible to affirm that, differently from what has been described for other vaccines, such pathological findings can persist even after 4 weeks. Considering the widespread use of US and its main role for lymph-node evaluation [12], it is essential for every radiologist to know how axillary, supra and subclavicular lymph nodes can appear at US after mRNA COVID-19 vaccination prior to assess potentially unnecessary and costly lymph node biopsies. In this context, further data are still necessary to establish the appropriate timeline of follow-up examinations, especially in oncologic patients. Mehta and colleagues proposed a follow-up range between 4 and 12 weeks, which might be appropriate [13]. However, with proper knowledge of US features and patient vaccination history, it has been hypothesized that follow-up could be unnecessary [11]. Interestingly, the recent recommendations suggest that imaging should be performed before vaccination whenever possible while nonurgent exams should be postponed for at least 6 weeks after the administration of the final dose [3]. However, re-scheduling diagnostic procedures might not be always easy and/or possible. Thus, radiologists should be aware of the incidence and significance of this side effect as well as be able to correctly assess its key imaging features. In the absence of a clear timing, a possibility could be to perform a US examination in selected cases before vaccine administration to rule out the presence of a pre-existing lymphadenopathy.

## Conclusion

In conclusion, mRNA-based vaccines can lead to the occurrence of pathological lymph nodes that can be indistinguishable from the metastatic ones at US. Although B-mode and color-Doppler features frankly mimic those of metastatic lymph nodes, quantitative parameters derived from shear-wave sonoelastography may be effective for the differential diagnosis. Future studies on larger cohorts of subjects should be conducted to further confirm our findings.

## Data Availability

The datasets used and/or analysed during the current study are available from the corresponding author on reasonable request. MATERIAL and data are all available.
